# Inhibition of ACVR1 in Cancer-Associated Fibroblasts Suppresses Colorectal Cancer Cell Growth

**DOI:** 10.1245/s10434-025-17765-0

**Published:** 2025-07-31

**Authors:** Shinya Kato, Norikatsu Miyoshi, Shiki Fujino, Masafumi Horie, Shinichi Yachida, Mitsunobu Takeda, Yuki Sekido, Tsuyoshi Hata, Atsushi Hamabe, Takayuki Ogino, Mamoru Uemura, Hirofumi Yamamoto, Masayoshi Yasui, Masayuki Ohue, Yuichiro Doki, Hidetoshi Eguchi

**Affiliations:** 1 Department of Gastroenterological Surgery, Graduate School of Medicine, Osaka University, Osaka, Japan; 2 Innovative Oncology Research and Regenerative Medicine, Osaka International Cancer Institute, Osaka, Japan; 3 Colorectal Surgery, Monash Health Dandenong Hospital, Dandenong, Australia; 4 Department of Molecular and Cellular Pathology, Graduate School of Medical Sciences, Kanazawa University, Kanazawa, Japan; 5 Department of Cancer Genome Informatics, Graduate School of Medicine, Osaka University, Osaka, Japan; 6 Department of Gastroenterological Surgery, Osaka International Cancer Institute, Osaka, Japan

**Keywords:** ACRV1, BMP7–ACRV1 axis, Cancer-associated fibroblast, Colorectal cancer, Organoid culture, Tumor growth inhibition, Two-dimensional organoid

## Abstract

**Background:**

Colorectal cancer (CRC) is the third most common malignancy worldwide and a leading cause of cancer-related mortality. Stromal signatures in CRC are correlated with poor prognosis and resistance to chemotherapy, affecting tumor progression and relapse. Although single-cell analyses have identified subpopulations of cancer-associated fibroblasts (CAFs), effective molecular targeted therapies against CAFs are lacking.

**Materials and Methods:**

We employed organoid culture methods, focusing on two-dimensional organoids (2DOs) to mimic CRC histology. Using single-cell analysis, we investigated cancer-fibroblast crosstalk, with emphasis on activin receptor type I (ACVR1) in fibroblasts and bone morphogenetic protein 7 (BMP7) in cancer cells as potential therapeutic targets. The correlation between high ACVR1 and BMP7 expression levels and the prognosis of patients with stage II cancer was evaluated.

**Results:**

The 2DO mouse xenograft model replicated the characteristics of the fibroblast subpopulations present in human CRC tumors. Single-cell RNA sequencing identified fibroblast clusters, with the BMP7–ACVR1 axis emerging as a potential therapeutic target. High BMP7 and ACVR1 expression was significantly correlated with poor disease-free survival and overall survival in stage II CRC. Administration of an ACVR1 inhibitor during the coculture of 2DOs and mouse stromal cells inhibited tumor growth.

**Conclusions:**

ACVR1 is a promising therapeutic target that inhibits CAF proliferation. High BMP7 and ACVR1 expression is a significant prognostic factor in stage II CRC.

**Supplementary Information:**

The online version contains supplementary material available at 10.1245/s10434-025-17765-0.

Colorectal cancer (CRC) is the third most prevalent malignancy and the fourth leading cause of cancer-related mortality.^1^ Stromal signatures of CRC correlate with poor prognosis and resistance to chemotherapy.^2^ Tumor stroma plays a critical role in tumor development, progression, drug resistance, and relapse.^3^ The microenvironment of the cancer stroma is composed of fibroblasts, immune cells, blood vessels, and the extracellular matrix (ECM).^4,5^ The fibroblasts surrounding cancer cells produce ECM, cytokines, proteases, and growth factors, functioning in either tumor promotion or suppression.^6,7^ In colorectal cancer, consensus molecular subtype 4 (CMS4), characterized by abundant fibroblast infiltration, is known to be associated with poor prognosis, and many studies have focused on the tumor-promoting functions of cancer-associated fibroblasts (CAFs).^8,9^ Recently, single-cell analyses identified certain subpopulations of CAFs.^10,11^ Different CAF populations have different effects on cancer growth. Previous lineage-tracking studies in mice reported that most of the alpha-smooth muscle actin (ACTA2 or αSMA)-expressing CAFs that act in a tumor-proliferative manner originated from local fibroblast proliferation^12,13^; however, effective molecular-targeted therapies for CAFs remain insufficiently developed.

To address the heterogeneity of cancers, investigators have increasingly used organoid culture methods. Patient-derived organoids retain the physiological characteristics of parent cancer cells and reflect the heterogeneity of disease subgroups.^14,15^ We have previously reported the development of two-dimensional organoids (2DOs) that closely resemble the histology of original CRC specimens from patients.^16,17^ By focusing on the phenomenon where 2DOs induce stromal cells in tumors xenografted subcutaneously into mice, we sought to elucidate cancer cell-fibroblast crosstalk using this model.

This study aimed to identify novel CAF markers that could serve as potential therapeutic targets. Using single-cell analysis, we demonstrated that the 2DO mouse subcutaneous xenograft model replicated aspects of the fibroblast subpopulations present in human colon cancer tumors. Additionally, we investigated cancer cell–fibroblast crosstalk, focusing on the expression of activin receptor type I (ACVR1) in fibroblasts and bone morphogenetic protein 7 (BMP7) in cancer cells as potential therapeutic targets. We examined the correlation between high expression levels of ACVR1 and BMP7 and prognosis in patients with stage II CRC.

## Materials and Methods

### Primary Culture of 2DOs

Primary cultures of iCC129 and iCC603 cells were established and cultured in a modified stem cell culture medium, according to previous reports.^16,17^ The cells were incubated under humidified atmosphere containing 5% CO_2_ at 37 °C, and the medium was changed every 3 days. Cells were harvested using Accutase (Nacalai Tesque, Kyoto, Japan).

### Establishment of DsRed-Expressing 2DOs

The vector pLV[Exp]-Neo-CMV>DsRed_Express2 (VB900088-2435mhv; Vector Builder, Chicago, IL, USA) was used to establish DsRed-expressing 2DOs. A high-titer lentiviral packaging mix with pLVSIN (Takara Bio, Kusatsu, Japan) was used to transfect the vector into 2DOs. DsRed-expressing cells were purified twice by single-cell sorting using an SH800 cell sorter (Sony, Tokyo, Japan). Sorted cells were cultured using the 2DO primary culture method described above.

### Xenograft Model

Accutase-dissociated 2DOs (5 × 10^5^ cells) suspended in Matrigel (BD Biosciences, Franklin Lakes, NJ, USA) were subcutaneously xenografted into the ventral flanks of 7- or 8-week-old nonobese diabetic/severe combined immunodeficient (NOD/SCID) mice (Clea, Tokyo, Japan). To isolate mouse stromal cells from the tumors, mice were sacrificed when the tumors reached a diameter of 10 mm. The Animal Research Committee of the Osaka International Cancer Institute approved the present study.

### Isolation and Culture of Mouse Stromal Cells (mSCs)

The tumor formed by the transplantation of DsRed-expressing 2DOs was resected, cut using a scalpel, and washed three times with normal saline (Otsuka Pharmaceutical Factory, Naruto, Japan). The tumors were minced to 1-mm pieces and dissociated with 1 mg/mL of collagenase (C6885; Sigma-Aldrich, St. Louis, MO, USA) in Dulbecco’s modified Eagle’s medium (Sigma-Aldrich), shaken using the gentleMACS^TM^ Octo Dissociator with Heaters (Miltenyi Biotec, Bergisch Gladbach, Germany) at 100 rpm for 20 min at 37 °C, and passed through a 200-μm filter (Sansho, Tokyo, Japan). Red blood cells in the solution were labeled with APC-Cy7 antimouse TER-119 (catalog number (cat. no.) 116223, Research Resource Identifier (RRID): AB_2137788, BioLegend, San Diego, CA, USA), and white blood cells were labeled with APC/Cyanine7 antimouse CD45 antibody (cat. no. 103116, RRID: AB_312981; BioLegend). The cells were then treated with 7-aminoactinomycin D (cat. no. 559925, RRID: AB_2869266; BD Biosciences) to remove dead cells. mSCs were sorted and obtained by negative selection of CD45 and DsRed using an SH800 cell sorter (Sony). mSCs were seeded in collagen-coated plates (4860-010; AGC Techno Glass, Shizuoka, Japan) and cultured in modified stem cell culture medium.

### Flow Cytometry

The expression of fluorescent-labeled surface proteins in dissociated tumor cells was analyzed using flow cytometry. The relative fluorescence intensities were measured using an SH800 cell sorter (Sony). The data were analyzed using FlowJo v10 (BD Biosciences).

### Patients and Clinical Tissue Samples

The present study included patients who underwent radical resection of CRC between 2009 and 2013 at the Osaka International Cancer Institute and were diagnosed with pathological stage II CRC. The mean age of the patients was 65.1 years (range 35–83), with 33 males and 30 females. Patient information was obtained from electronic medical records. Pathological factors were determined according to the eighth edition of the Union for International Cancer Control Tumor–Node–Metastasis Classification. The resected specimens were fixed in formaldehyde, dehydrated in ethanol, embedded in paraffin, sliced to 3 μm, and stained with hematoxylin–eosin (HE) and Elastica van Gieson. The stroma of the tumor-invading areas was identified via HE staining. Enrolled patients were followed up through medical interviews, physical examinations, and tumor marker assessments every 3 months, along with abdominopelvic computed tomography (CT) scans every 6 months. To evaluate for anastomotic recurrence, colonoscopic examinations were performed at 1 and 3 years postoperatively. Patients underwent surveillance for approximately 5 years after surgery. In cases where recurrence was suspected, positron emission tomography/computed tomography was conducted at the discretion of the attending physician. 

All participants provided informed consent for the study and publication. This study was approved by the Institutional Review Board of Osaka International Cancer Institute (no. 1801129300-4 and no. 1711015224-25).

### Immunohistochemistry and Immunofluorescence

Immunohistochemistry was performed by deparaffinization and blocking. Sections were incubated with primary anti-ACVR1 rabbit antibody (cat. no. HPA007505, RRID: AB_1844752; Sigma-Aldrich), anti-BMP7 rabbit polyclonal antibody (cat. no. PA5-11720, RRID: AB_2063865; Thermo Fisher Scientific, Waltham, MA, USA) at a dilution of 1:100, anti-cytokeratin 20 mouse antibody (cat. no. ab854, RRID: AB_2133708; Abcam, Cambridge, UK), and anti-alpha smooth muscle actin antibody (cat. no. ab5694, RRID: 2223021; Abcam) at a dilution of 1:200 overnight at 4 °C. After incubation with a biotinylated secondary antibody for 30 min, the sections were incubated with the ImmPACT DAB Kit (Vector Laboratories, Burlingame, CA, USA) for 1.5 min. Sections were incubated with fluorescent secondary antibodies (goat antirabbit immunoglobulin (Ig)G Alexa Fluor 488, cat. no. A11008, RRID: AB_143165, Thermo Fisher Scientific; goat antimouse IgG Alexa Fluor 594, cat. no. A11005, RRID: AB_2534073, Thermo Fisher Scientific) at a dilution of 1:2000 for 30 min, followed by incubation with Prolong DAPI (P36931, Thermo Fisher Scientific). ImageJ (version 2.9.0/1.53t) was used for intercellular distance analysis. A total of three high-power fields of the stroma in the tumor invasion area were assessed and scored (0–2 points), and the points were summed; high expression was defined as 4–6 points and low expression as 0–3 points.

### Direct Coculture

2DOs (3 × 10^3^ cells/well) were seeded into each well of a 96-well plate. The mSCs (1 × 10^3^ cells/well) were seeded in the same dish as the direct coculture group. The proliferation of the organoids was assessed by measuring the area of DsRed fluorescence using an all-in-one fluorescence microscope (BZ-X700; Keyence, Osaka, Japan).

### Indirect Coculture

For indirect coculture, 25% iCC129 and iCC603-conditioned medium (ICM) was first obtained from the supernatant of the medium at 48 h after seeding of iCC129 and iCC603 cells by filtration through a 0.45-µm filter. The mSCs (3000 cells) were seeded in each well of a 96-well plate. The coordinates of three low-power fields of view per well were recorded after cell adhesion. The medium was replaced with either normal stem cell culture medium or with the ICM for the negative control and ICM groups, respectively, 24 h after seeding. The number of mSCs in each field of view was evaluated over time after changing the medium.

### Drug Sensitivity for 5-FU

2DOs (1 × 10^3^) were seeded in 96-well plates and incubated for 48 h before 5-FU (068-01401, Wako Pure Chemical Industries, Osaka, Japan) administration. Cell proliferation in distilled water (the drug diluent) was used as the control. The ratio of the number of viable adhered cells after drug administration to that of the control was determined. A total of three independent experiments were performed and the average values are presented. The formula used for the calculation is as follows: 100 × Control 0 h cell number × Drug 96 h cell number / {(Control 96 h cell number − Control 0 h cell number) × Drug 0 h cell number}. The number of viable cells in each well was determined by measuring the area of DsRed fluorescence using the All-in-One fluorescence microscope (BZ-X700, Keyence)

### qRT-PCR

Total RNA was extracted using an RNA Purification Kit (Qiagen, Santa Clarita, CA, USA). Quantitative analysis was conducted through RT-PCR utilizing 100 nmol/L universal probe libraries, 0.1× FASTStart TaqMan Probe Master (Roche Diagnostics, Rotkreuz, Switzerland) for custom primers, iTaq Universal SYBR Green Supermix (Bio-Rad, Hercules, CA, USA) for commercially available primers, and 100 nmol/L primers with 10 ng of cDNA for target gene amplification. PCR was performed in a 96-well plate with 20 μL of master mix per well, and signals were detected using the CFX Connect Real-Time PCR Detection System (Bio-Rad). The thermocycling conditions were set to 1 cycle at 95 °C for 10 min, followed by 40 cycles at 94 °C for 10 sec, 60 °C for 20 sec, and 72 °C for 1 sec. Primers are detailed in Supplementary Table S1.

### Single-Cell RNA Sequencing

Single-cell library preparation was carried out in accordance with the manufacturer’s protocol for the Chromium Next GEM Single Cell 3′ Reagent Kits (v3.1; 10× Genomics, Pleasanton, CA, USA), and the resulting libraries were sequenced using a HiSeq X sequencer (Illumina, San Diego, CA, USA). The data matrix was generated through Cell Ranger pipeline (version 6.1.2). Raw reads were mapped to the human reference genome (GRCh38) and mouse reference genome (Mm10) via the STAR aligner. Seurat software (version 4.1.0) was employed for quality control and downstream analyses. Low-quality cells were excluded on the basis of the following criteria: nFeature_RNA 1000–7000 and mitochondrial gene content below 15%. Ultimately, 2207 human cancer cells and 589 mouse stromal cells that passed quality control were retained for further investigation. Dimensionality reduction analysis was conducted using uniform manifold approximation and projection (UMAP) at a resolution of 0.8. Marker genes distinguishing the different clusters were identified using the FindAllMarkers function. Cell-to-cell communication between human cancer and mouse stromal cells was assessed using CellPhoneDB.^18^

### Statistical Analysis

Continuous variables are expressed as means and standard errors. Student’s *t*-test was used to analyze the differences between two independent groups. Continuous variables exhibiting a nonparametric distribution within the population were assessed using the Mann–Whitney *U* test. Differences in clinicopathological characteristics between the two groups were evaluated with Fisher’s exact test. Kaplan–Meier survival curves were generated and compared via the generalized log-rank test. All analyses were conducted using JMP Pro 17 (SAS Institute), and statistical significance was defined as *p* < 0.05.

## Results

### mSCs Isolated from Xenografted Tumors Promote Proliferation of Patient-Derived Organoids in Coculture

**Fig. 1:**
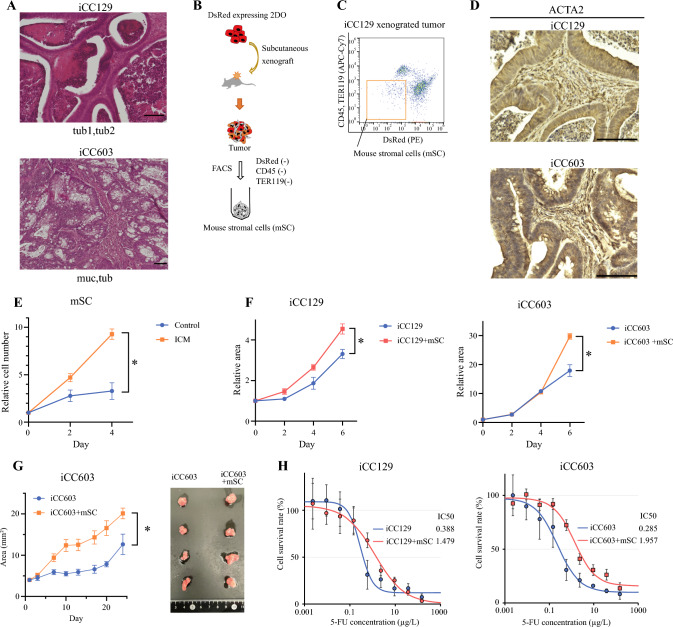
Coculture of 2DOs and mSCs from 2DO-xenografted tumors; **A** representative histological examinations of xenografted tumors from 2DOs; **B** strategy for mSC isolation; **C** representative gating image of isolating CD45^−^TER119^−^DsRed^−^ cells via fluorescence-activated cell-sorting is shown for 2DO xenograft tumor; **D** representative ACTA2 immunohistochemistry images of xenografted tumors from 2DOs; **E** proliferation assay of mSCs (*N* = 4) when indirectly cocultured with 2Dos; *ICM* 25% iCC129-conditioned medium; **F** proliferation of iCC129 and iCC603 cells (*N* = 4) when directly cocultured with mSCs; **G** comparison of tumor proliferation in vivo (*N* = 4) between 2DOs alone and 2DOs cocultured with mSCs; **H** comparison of drug sensitivity to 5FU (*N* = 4) between 2DOs alone and 2DOs cocultured with mSCs; data are presented as the mean ± standard error of the mean; *p < 0.05

Histological analysis of tumors formed by the subcutaneous implantation of DsRed-expressing 2DOs into NOD/SCID mice revealed a resemblance to the patient specimens (Fig. 1A). The subcutaneous tumors were homogenized, and DsRed^−^CD45^−^ and TER119^−^ cells were sorted by FACS to isolate mSCs (Fig. 1B, C). Unlike the 2DOs used (iCC129 and iCC603 cells), the mSCs expressed Acta2 and Vim, a mesenchymal marker (Supplementary Fig. S1). Immunohistochemical staining of the subcutaneous tumors also showed high stromal ACTA2 expression (Fig. 1D).

mSCs cultured in ICM exhibited a significantly higher proliferative capacity than mSCs cultured in normal medium (Fig. 1E). The cancer cells and mSCs were cocultured in the same dish to evaluate cancer cell proliferation. 2DOs cocultured with mSCs showed enhanced proliferation compared with 2DOs cultured alone (Fig. 1F). Furthermore, we assessed the in vivo proliferative potential of 2DOs subcutaneously transplanted into mice and compared it with that of a mixture of 2DO and mSCs transplanted into mice. The implanted tumors with a mixture of 2DO and mSCs exhibited accelerated growth compared with the implanted 2DO alone. Conversely, subcutaneous transplantation of mSCs alone did not result in tumor formation (Fig. 1G). 2DOs cocultured with mSCs showed reduced drug sensitivity to 5-FU compared with 2DOs cultured alone (Fig. 1H).

### The Stroma of 2DO-Xenografted Tumors Partially Replicates the Gene Expression Patterns Observed in the Stroma of CRC

**Fig. 2:**
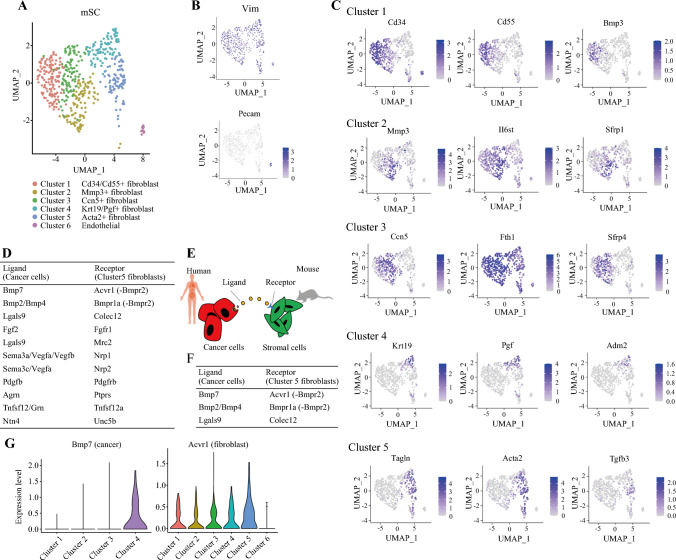
Single-cell RNA sequencing analysis of 2DO xenografted tumors; **A** cluster analysis of mSCs within 2DO xenografted tumors using UMAP; **B** distribution of Vim and Pecam1 expression among mSCs; **C** representative gene expression profiles for each cluster; **D**, **E** list of upregulated receptors in Cluster 5 fibroblasts and their corresponding ligands expressed in 2DOs; **F** ligand–receptor pairs upregulated in 2DOs and fibroblasts that are correlated with patient prognosis in the TCGA database; **G** expression of BMP7 in cancer cells and ACVR1 in fibroblasts across clusters

Single-cell RNA sequencing was performed on the 2DO xenograft tumors. The genetic sequences of the species and the DsRed clearly distinguished cancer cells from noncancerous cells. The mSCs clustered into six subgroups (Fig. 2A, C). Most stromal cells were positive for vimentin, a mesenchymal marker. Cluster 6 consisted of a CD31-positive vascular endothelial cell population (Fig. 2B). The gene expression profiles of each mSC cluster were compared with those of the CAF clusters identified in a previous single-cell analysis^19,20^ of a large human colon cancer cohort. The expression profile of cluster 5 was equivalent to that of myofibroblast-like CAFs, as reported by Öhlund et al. ^10^ This corresponds to cS26 (myofibroblasts) and cS27 (CXCL14^+^ fibroblasts) as reported by Pelka et al. ^19^ Cluster 2 exhibited an upregulation of inflammation-related genes, such as Mmp3 and Il6st, and was considered to correspond to the inflammatory CAFs identified by Öhlund et al. ^10^ This corresponds to cS29 (MMP3^+^ CAF), as reported by Pelka et al. ^17^ (Fig. 2D, E). A total of three gene combinations associated with poor prognosis, due to their high expression in the The Cancer Genome Atlas (TCGA) database and lack of previously reported markers for cancer stroma, were selected as potential therapeutic targets (Fig. 2F, G). We focused on one of these combinations, BMP7 in cancer cells and ACVR1 in stromal cells.

### ACVR1 Inhibitor Suppresses Cancer Cell Proliferation When Cocultured with Stromal Cells

**Fig. 3:**
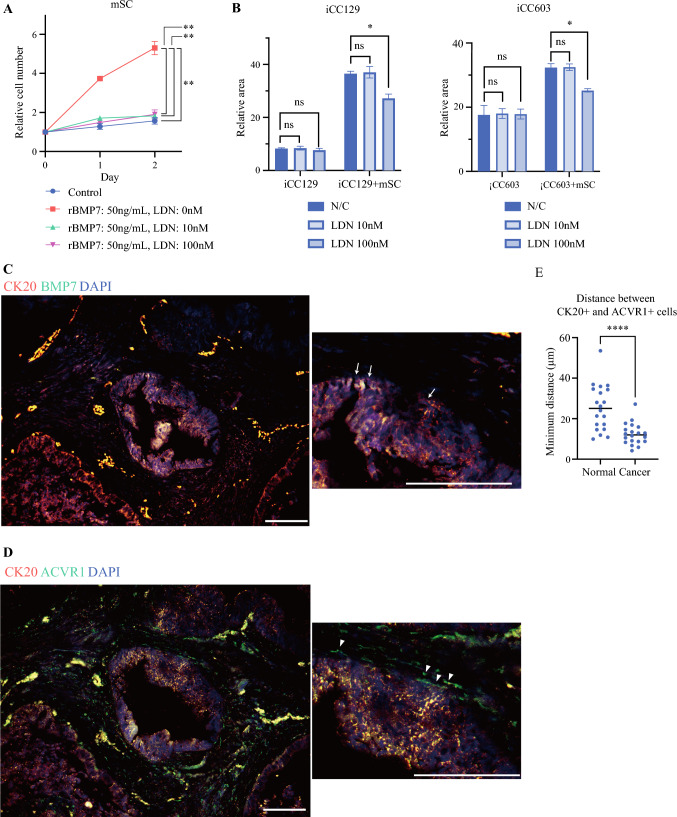
Effect of ACVR1 inhibitor (LDN193189) on 2DOs and mSCs; **A** proliferation curves of mSCs to assess the response to LDN193189 upon administration of rBMP7; **B** proliferation of 2DOs under administration of LDN193189; (C, D) representative images of double immunofluorescence assay results using specimens from patients with colorectal cancer; these are continuous sections; **C** CK20, BMP7, and DAPI are stained red, green, and blue, respectively; arrows indicate representative CK20^+^BMP7^+^ cells (yellow–white); **D** CK20, ACVR1, and DAPI are stained red, green, and blue, respectively; arrowheads indicate representative CK20^−^ACVR1^+^ cells (green); **E** comparison of minimum distance between CK20^+^ACVR1^−^ cells and CK20^−^ACVR1^+^ cells in invasive area of colorectal cancer and normal colon duct (*N* = 20); data are presented as the mean ± standard error of the mean: scale bar: 100 μm; *p < 0.05, **p < 0.01, ***p < 0.001

The proliferation of mSCs treated with BMP7 and their response to the ACVR1 inhibitor (LDN-193189) upon BMP7 administration were evaluated. BMP7 significantly promoted mSC proliferation, while LDN-193189 inhibited this effect (Fig. 3A). LDN-193189 alone did not inhibit the proliferation of 2DOs (Fig. 3B). However, it did inhibit the proliferation of 2DOs cocultured with mSCs (Fig. 3B). This suggests that LDN-193189 indirectly inhibited 2DO growth by targeting mSC proliferation.

The BMP7 and ACVR1 expression sites in advanced tumors were visualized using double immunofluorescence (Fig. 3C, D). CK20 serves as a surrogate marker within the cancerous region, with BMP7 predominantly coexpressed in CK20-positive cells. The minimum distance between CK20^+^ACVR1^−^ and CK20^−^ACVR1^+^ cells was compared between the invasive areas of CRC and the normal colorectal glandular ducts within the same sections (Fig. 3E, Supplementary Fig. S2). This distance was significantly reduced in the cancerous region, suggesting that BMP7-positive cancer cells and ACVR1-positive stromal cells interacted within the stromal area near the advanced cancer invasion front.

### High Expression of Oncogenic BMP7 and Stromal ACVR1 in Advanced Tumor Regions Is Associated with Poor Prognosis in Stage II CRC

**Fig. 4:**
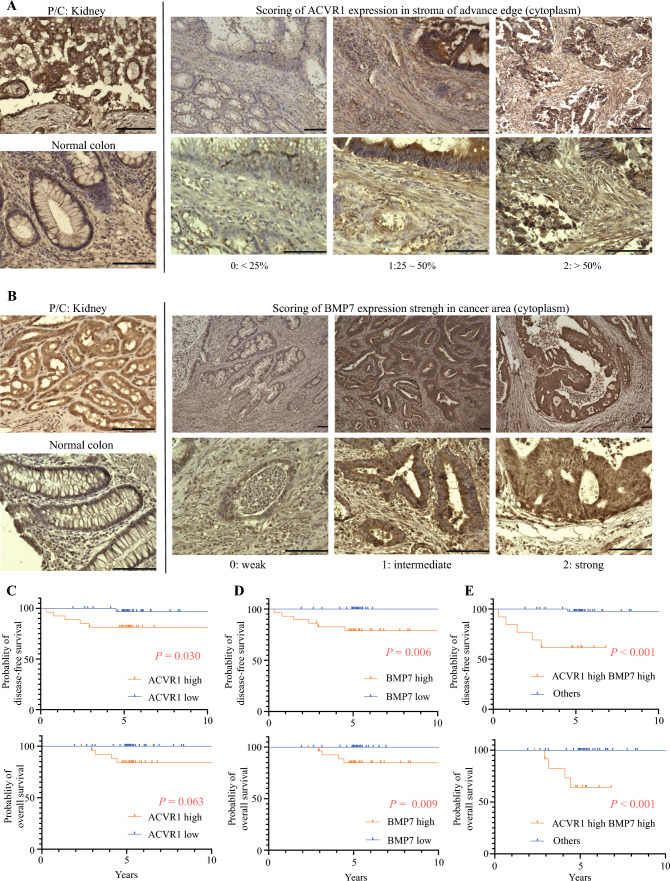
High expression of oncogenic BMP7 and stromal ACVR1 in advanced tumor regions is associated with poor prognosis in stage II colorectal cancer.; **A** representative ACVR1 immunostaining section in stage II CRC resected specimen of invasive margin; **B** representative BMP7 immunostaining section in stage II CRC resected specimen of cancerous area (C, D) Kaplan–Meier curves for DFS and OS based on **C** ACVR1 and **D** BMP7 expression; **E** Kaplan–Meier curves for DFS and OS based on high BMP7 and high ACVR1 expression in patients or otherwise; Scale bar: 100 μm

The expression of BMP7 in cancerous regions and ACVR1 in stromal regions of resected stage II CRC specimens was evaluated by immunohistological staining. To investigate the association between tumor–stroma interactions and patient prognosis, we conducted the analysis in patients with stage II CRC. A total of 63 patients with stage II CRC who underwent radical resection between 2009 and 2013 at the Osaka International Cancer Institute and 63 patients with stage II CRC were included (Table 1). High stromal ACVR1 expression in areas of cancer invasion was significantly correlated with poor disease-free survival (DFS) (Fig. 4A, C and Supplementary Table S2). Similarly, high BMP7 expression in cancerous regions was significantly associated with poor prognosis in both DFS and overall survival (OS) (Fig. 4B, D and Supplementary Table S3). Patients exhibiting high BMP7 expression in the cancerous area and high ACVR1 expression in the stromal area had markedly poorer OS and DFS rates than the other patients (Fig. 4E, Table 1, and Supplementary Table S4).

**Table 1:** Clinicopathological factors of patients with stage II colorectal cancer

Factor	ACVR1 high BMP7 high (*n* = 13)	Others (*n* = 50)	*p*-Value
Age, years (median, IQR)	69 (58.5–73)	57 (57–75)	0.980^a^
Sex (male, female)	8 (61.5%)/5 (38.5%)	25 (50.0%)/25 (50.0%)	0.542^b^
Colon/rectum	6 (46.2%)/7 (53.8%)	32 (64.0%)/18(36.0%)	0.341^b^
Tumor invasion (T3/T4)	6 (46.2%)/7 (53.8%)	30 (60.0%)/20 (40.0%)	0.531^b^
Lymphatic invasion (absent/present)	7 (63.6%)/6 (36.4%)	31 (62.0%)/19 (38.0%)	0.752^b^
Vascular invasion (absent/present)	3 (23.1%)/10 (76.9%)	16 (32.0%)/34 (68.0%)	0.738^b^

^a^Mann–Whitney *U* test, ^b^Fisher’s exact test, *IQR* interquartile range

## Discussion

The stroma formed in 2DO xenograft tumors in this study partially reproduced the heterogeneity of gene expression in the stroma formed in patients with colon cancer. Using ligand–receptor analysis of reproducible fibroblast clusters, we focused on the cancer cell BMP7–fibroblast ACVR1 axis. The results suggest a potential treatment for CAFs. Additionally, elevated expression of ACVR1 was observed in the stroma adjacent to the advanced regions of the tumor compared to that in the normal colon epithelium.

However, the origin and contribution of CAFs remain unclear. Bone marrow transplantation experiments in a mouse model of gastric cancer showed that 20% of ACTA2^+^ CAFs were recruited from the bone marrow. In contrast, Acta2-RFP^+^ CAFs were not detected in small intestinal tumors developed in an ApcMin/+ parabiosis study using Acta2-RFP mice.^12^ Previous lineage-tracking studies have shown that approximately 75% of ACTA2^+^ CAFs in CRC are generated by local fibroblast proliferation, with the remaining 25% acquired through novel or conserved ACTA2 expression in existing fibroblasts.^13^ In addition, human studies using secondary tumors that developed after sex-mismatched bone marrow transplantation have shown that bone marrow-derived cells are not the primary cause of ACTA2^+^ CAFs.^21,22^ Therefore, it is important to control the supply of CAFs via local fibroblast proliferation in CRC, although the origin and contribution of CAFs vary according to carcinoma, context, and tumor stage.

BMPs are extracellular multifunctional signaling cytokines, part of the transforming growth factor β (TGF-β) superfamily.^23^ Binding of BMP ligands, such as BMP2, 4, and 7 to type I and type II BMP receptors induces the phosphorylation of SMAD1/5/8, which binds to SMAD4 and increases the expression of target genes such as ID1, 2, 3, and 4.^24^ In the normal colon, the epithelial stem cell niche is maintained by low BMPs and high Wnt at the base of the crypt; however, epithelial cell differentiation is driven by increased BMPs and low Wnt toward the luminal surface.^25^ However, the role of BMP signaling in cancer remains unclear. Several studies have shown that BMP signaling plays a role in tumor delay in CRC cells themselves.^26–29^ By contrast, BMP signaling has recently been reported to promote the growth of xenograft tumors derived from primary human CRC, and autocrine BMP4 signaling has been identified as a therapeutic target in CRC.^30,31^ These controversies may be attributed to the heterogeneity of cancer and its microenvironment.

Shimizu et al. ^32^ showed that BMP inhibitors can suppress the growth of specific populations of CRC organoids. For the organoids used in this study, the effect of the BMP inhibitors was not apparent when the organoids were cultured alone. However, BMP inhibitors significantly suppressed tumor growth when cocultured with stromal cells. It is highly likely that the inhibition of tumor-promoting CAF growth suppressed TGF-β or other substances secreted by the CAFs (Supplementary Fig. S3), ^13,33,34^ leading to the inhibition of CRC organoid growth. BMP inhibitors may also inhibit tumor growth within the tumor microenvironment, even in CRC organoid populations that were previously shown to be resistant to BMP inhibitors. ACVR1 inhibitors may hold therapeutic potential as treatment agents targeting the tumor stroma in colorectal cancer; however, BMPs regulate tissue homeostasis by acting on Lgr5⁺ stem cells in the intestinal epithelium. Therefore, in vivo administration of ACVR1 inhibitors may impair intestinal regeneration, warranting careful consideration.^35^

One limitation of the current study is that because it is a xenograft model utilizing immunosuppressed mice, the immune response to the tumor was disregarded, rendering the model an incomplete mimic of a patient’s CRC. In addition, because this is a xenograft model, it would be challenging to reproduce gene expression that perfectly matches that of the human CRC stroma. However, the organoid xenograft model employed in this study is considered a relatively straightforward method for replicating gene expression in colon CAFs within the tumor microenvironment. Another limitation is that, while the study demonstrated that administering an ACVR1 inhibitor during the coculture of 2DOs and mouse stromal cells inhibited tumor growth, it did not investigate the dynamics of gene expression in the CAFs and 2DOs.

In conclusion, this study demonstrated that ACVR1 is a potential therapeutic target that inhibits the proliferation of CAFs and ACVR1-positive stromal cells in advanced tumor stroma adjacent to tumor cells. Furthermore, it was shown that BMP7 and ACVR1 were significant prognostic factors for OS and DFS of patients with stage II CRC. In the future, strategies that control tumor progression by regulating stromal growth may hold significant clinical value in the treatment of CRC.

## Supplementary Information

Below is the link to the electronic supplementary material.

Supplementary file1 (DOCX 10275 KB)

## Data Availability

scRNA-seq data are available from the corresponding authors upon reasonable request.
