# Supplementary material for: Inhibition of ACVR1 in Cancer-Associated Fibroblasts Suppresses Colorectal Cancer Cell Growth
Source: Ann Surg Oncol. 2025 Jul 31;32(10):7333–43. doi: 10.1245/s10434-025-17765-0 (PMC12454473; doi:10.1245/s10434-025-17765-0)
Supplement: Supplementary file 1 — Supplementary file1 (DOCX 10275 KB) [file 10434_2025_17765_MOESM1_ESM.docx]

**Supplementary Figure S1**

**Gene expression of mesenchymal markers in mSCs and 2DO**


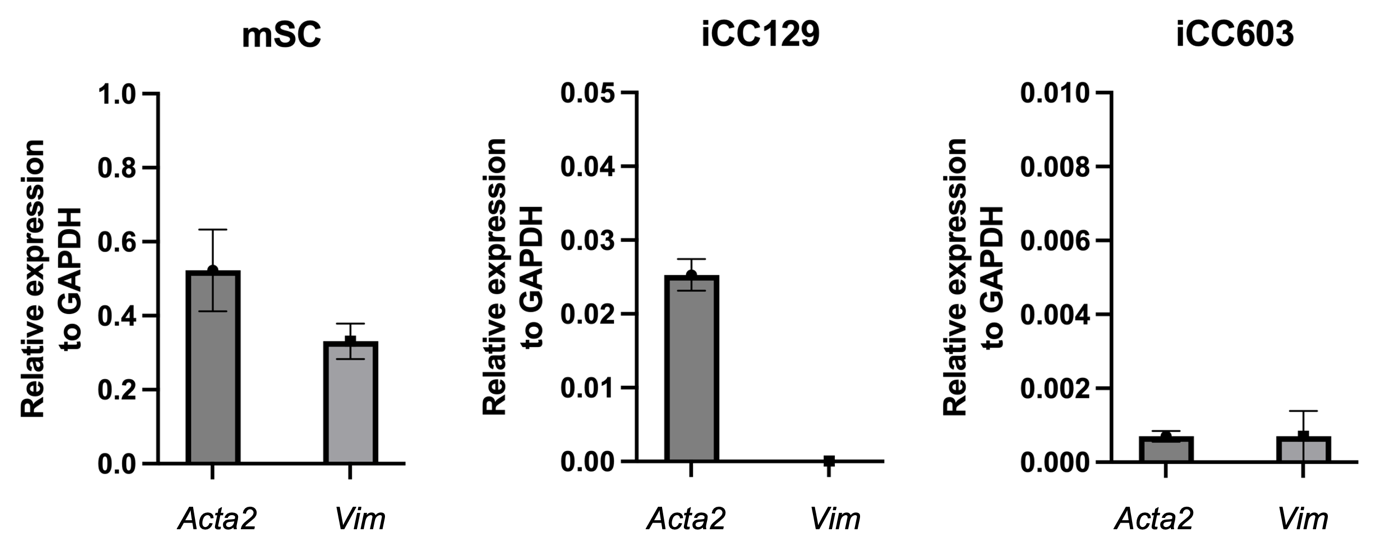


Relative expression of *Acta2* and *Vim* to Gapdh in mSCs and 2DOs by RT-PCR are shown.

**Supplementary Figure S2**

**Representative images of distance measurement between CK20^+^ACVR1^–^ cells and CK20^–^ACVR1^+^ cells in invasive area of colorectal cancer and normal colon duct**


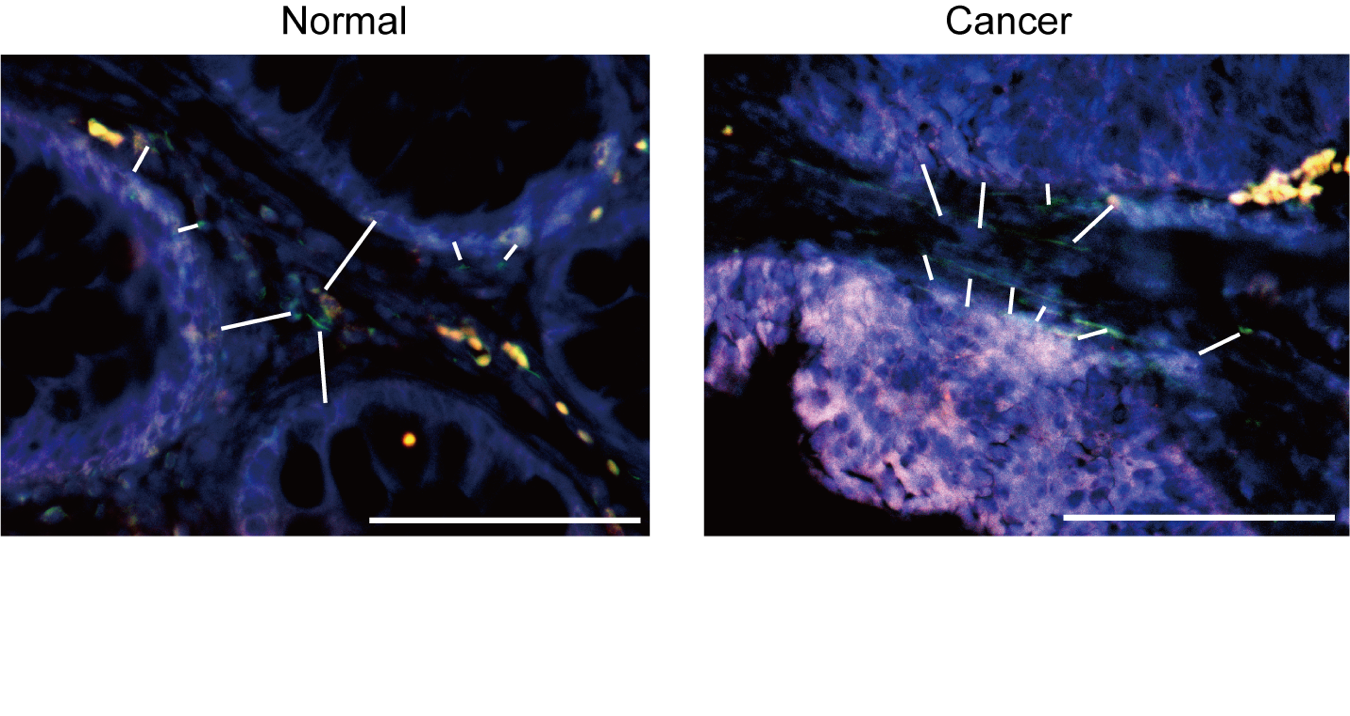


White lines indicate minimum distance between CK20^+^ACVR1^–^ cells and CK20^–^ACVR1^+^ cells. Scale bar: 100μm.

**Supplementary Figure S3**

**Tgfb1 and Tgfb3 expression in fibroblasts using single-cell RNA sequencing analysis**


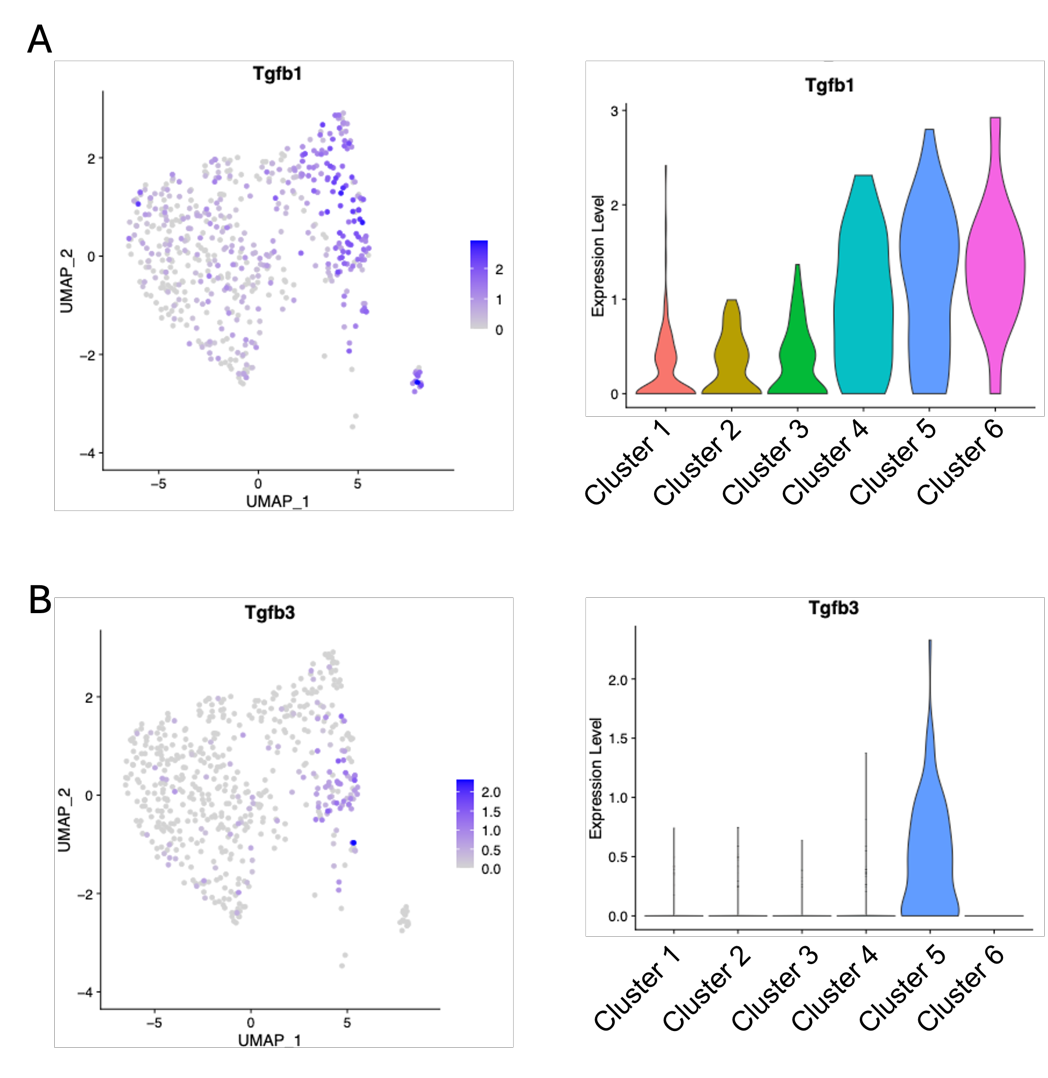


Tgfb1 (A) and Tgfb3 (B) expression in fibroblasts across clusters. They are upregulated in cluster 5 (Acta2^+^ fibroblasts) in 2DO xenografted tumors.

**Supplementary Table S1**

**PrimePCR SYBR Assay (Bio-Rad, Hercules, CA, USA)**

| Gene | Product No. |
| --- | --- |
| GAPDH, Human | 10025637 |
| GAPDH, Mouse | 10025637 |
| ACTA2, Human | 10025636 |
| ACTA2, Mouse | 10025636 |
| VIM, Human | 10025636 |
| VIM, Mouse | 10025636 |

**Supplementary Table S2**

**Comparison of clinicopathological factors between patients with colorectal cancer of ACVR1 high and low**

| Factor | ACVR1 high (n=27) | ACVR1 low (n=36) | p value |
| --- | --- | --- | --- |
| Age, years (median, IQR) | 66 (58–75) | 64 (55.5–75) | 0.917^a^ |
| Sex (male, female) | 18 (66.7%) / 9 (33.3%) | 15 (41.7%) / 21 (58.3%) | 0.074^b^ |
| Colon / rectum | 14 (51.9%) / 13 (48.1%) | 24 (66.7%) / 12 (33.3%) | 0.301^b^ |
| Tumor invasion (T3/T4) | 12 (44.4%) / 15 (55.6%) | 24 (66.7%) / 12 (33.3%) | 0.122^b^ |
| Lymphatic invasion (absent/present) | 17 (63.0%) /10 (37.0%) | 21 (58.3%) / 15 41.70%) | 0.798^b^ |
| Vascular invasion (absent/present) | 7 (25.9%) / 20 (74.1%) | 12 (33.3%) / 24 (66.7%) | 0.588^b^ |

IQR, interquartile range.

^a^Mann-Whitney U test, ^b^Fisher’s exact test.

**Supplementary Table S3**

**Comparison of clinicopathological factors between patients with colorectal cancer of BMP7 high and low**

| Factor | BMP7 high (n =29) | BMP7 low (n =34) | p value |
| --- | --- | --- | --- |
| Age, years (median, IQR) | 61 (55–73) | 69 (59.5–75.8) | 0.145^a^ |
| Sex (male, female) | 14 (48.3%) / 15 (51.7%) | 19 (55.9%) / 15 (44.1%) | 0.617^b^ |
| Colon / rectum | 16 (55.2%) / 13 (44.8%) | 22 (64.7%) / 12 (35.3%) | 0.606^b^ |
| Tumor invasion (T3/T4) | 13 (44.8%) / 116 (55.2%) | 23 (67.7%) / 11 (32.3%) | 0.080^b^ |
| Lymphatic invasion (absent/present) | 14 (48.3%) / 15 (51.7%) | 24 (70.6%) / 10 (29.4%) | 0.120^b^ |
| Vascular invasion (absent/present) | 9 (31.0%) / 20 (69.0%) | 10 (29.4%) / 24 (70.6%) | 0.999^b^ |

IQR, interquartile range.

^a^Mann-Whitney U test, ^b^Fisher’s exact test.

**Supplementary Table S4**

**Details regarding patients with recurrence**

| No | Age | Sex | ACVR1 high/low | BMP7 high/low | Site of recurrence |
| --- | --- | --- | --- | --- | --- |
| 1 | 71 | Male | high | high | Liver |
| 2 | 73 | Male | high | high | Liver, lung, local recurrence |
| 3 | 55 | Male | low | high | Lung (multiple metastasis) |
| 4 | 70 | Male | high | high | Liver (multiple metastasis) |
| 5 | 55 | Female | high | high | Lung (multiple metastasis) |
